# Study on the Correlation Between GDF-15 Levels and a Diagnostic Model for Diabetic Retinopathy

**DOI:** 10.1155/jdr/6959604

**Published:** 2025-09-18

**Authors:** Xue-Nan Lian, Xian-Ling Zheng, Ming-Ming Zhu, Zong-Hu Li

**Affiliations:** ^1^School of Graduate Studies, Hebei North University, Zhangjiakou, China; ^2^Department of Endocrinology, Handan Central Hospital, Handan, China; ^3^Physical Examination Center, Handan Central Hospital, Handan, China

**Keywords:** apolipoprotein A1, diabetes, diabetic retinopathy, growth differentiation factor-15, macrophage inhibitory cytokine-1

## Abstract

**Background and Purpose:** Diabetic retinopathy (DR) is a chronic complication that affects approximately one-third of individuals with diabetes and represents a serious threat to vision. In recent years, increasing attention has been given to biomarkers and cytokines related to inflammation for their roles in disease mechanisms. This study is aimed at investigating the association between growth differentiation factor-15 (GDF-15) and the risk of DR in Handan, China, and developing a predictive model based on patients' clinical characteristics.

**Methods:** Between January and July 2024, patients with Type 2 diabetes mellitus (T2DM) treated at Handan Central Hospital were enrolled and classified into two groups: 74 patients without DR (NDR) and 79 with DR. Stepwise regression was used to select variables, and a logistic regression model was constructed to predict the risk of DR. Additionally, 17 healthy individuals (control group, CG) were included to explore GDF-15 distribution across different populations.

**Results:** Compared to the NDR group, patients with DR showed significantly lower levels of HB, ALB, CO_2_, and 2h-CP and considerably higher levels of DvT, UREA, HDL-C, ApoA-1, and GDF-15. A logistic regression model incorporating six key variables—ALB, ApoA-1, CO_2_, DvT, 2h-CP, and GDF-15—was developed, yielding an accuracy of 0.936 (95% CI: [0.786, 0.992]), which outperformed the model based solely on GDF-15. Comparison among the three groups showed that GDF-15 levels were highest in the DR group and increased progressively with diabetes severity.

**Conclusion:** GDF-15 levels are significantly associated with the presence and progression of DR. The logistic regression model demonstrates high predictive value, suggesting that GDF-15 may serve as a promising biomarker for the early diagnosis and intervention of DR.

## 1. Introduction

Globally, the prevalence of DR is approximately 22.27% [1], while in China, it is reported to be 16.3%, posing a significant threat to vision [2]. Studies have shown that in patients with Type 2 diabetes, obesity and the progressive activation of adipose tissue macrophages (ATMs) contribute to insulin resistance and chronic tissue inflammation [3]. In addition, disruption of the intestinal barrier and an increase in proinflammatory bacteria are commonly observed in diabetes, contributing to gut microbiota dysbiosis [4]. In individuals with diabetes, elevated plasma glucose levels, combined with stimulation by reactive oxygen and nitrogen species, increase glucose flux in retinal cells, making capillary endothelial cells more susceptible to damage [5]. DR is now recognized as both a vascular and neurodegenerative disease, with clinical manifestations that emerge as diabetes progresses. In the early stages of retinal microvascular disease, metabolic dysregulation of lipids and glucose leads to sustained leukocyte activation, capillary occlusion, and retinal ischemia, ultimately culminating in the clinical signs observed in the later stages of DR [3].

There is a growing interest in the role of inflammatory markers and risk factors associated with endothelial dysfunction in DR, particularly cytokines [6]. GDF-15, also known as macrophage inhibitory cytokine-1 (MIC-1), is expressed in macrophages, endothelial cells, and adipocytes in response to various stressors [7]. MIC-1 mRNA has been detected in both humans and mice and is positively correlated with adiponectin mRNA expression, promoting adiponectin secretion by adipocytes and confirming the role of GDF-15 as an adipokine [8]. In murine models, GDF-15 has been shown to suppress appetite, increase energy expenditure and thermogenesis, promote lipolysis and oxidative metabolism, reduce body weight, and enhance insulin sensitivity and glucose homeostasis—highlighting its potential as a novel therapeutic target [9]. However, elevated levels of GDF-15 have also been associated with an increased risk of cardiovascular events [10]. Notably, individuals with obesity or T2DM exhibit significantly higher GDF-15 levels compared to healthy controls [11]. GDF-15 has been implicated in the development and progression of cardiovascular disease, diabetes, and cancer [12] and is currently used as a predictive biomarker for the onset of proteinuria in diabetic nephropathy (DN) [13].

Additionally, studies have shown that GDF-15 expression in patients with T2DM is significantly associated with both the number and function of circulating angiogenic endothelial progenitor cells [14], and its levels are elevated in inflammatory vitreoretinopathy [15]. Given that both DN and DR are microvascular complications of diabetes, a positive correlation between GDF-15 and DR has also been reported [16, 17]. However, the precise nature of this association remains unclear. As GDF-15 continues to gain clinical relevance, our study further investigated its correlation with the risk of DR and developed a diagnostic model based on T2DM patients in the Handan region.

## 2. Subjects and Methods

### 2.1. Subjects

A total of 153 patients with T2DM were admitted to Handan Central Hospital between January and July 2024. These patients were divided into two groups: 74 patients with NDR and 79 patients with DR. Additionally, a control group consisting of 17 CG was included. Clinical examinations and biochemical parameters were recorded and compared between the DR and NDR groups. Furthermore, correlations between these parameters—including GDF-15 levels—and the risk or protective factors for DR were analyzed.

### 2.2. Methods

#### 2.2.1. Data Collection

Basic demographic information and biochemical parameters were collected from all participants. Peripheral venous blood samples were obtained following a minimum fasting period of 8 h. Fundus photographs were captured using a nonmydriatic fundus camera.

#### 2.2.2. Diabetes Diagnostic Criteria

The diagnosis of diabetes was made according to the criteria established by the World Health Organization (WHO) in 1999. DR was graded based on the 2002 International Clinical Diabetic Retinopathy Disease Severity Scale. The Ethics Committee of Handan Central Hospital approved all experimental protocols. The study was conducted by the Declaration of Helsinki and relevant national guidelines and regulations. Informed consent was obtained from all participants before their inclusion in the study.

#### 2.2.3. Inclusion and Exclusion Criteria

Inclusion criteria are as follows:

1. Diagnosed with Type 2 diabetes according to the 1999 WHO criteria.
2. Availability of complete clinical data.
3. Informed consent provided by the patient.

Exclusion criteria are as follows:

1. Type 1 diabetes or gestational diabetes.
2. Presence of acute complications of diabetes.
3. Other forms of retinopathy or unclear fundus images.
4. Malignant tumors, thyroid disorders, or hematological diseases.
5. Acute or chronic pancreatitis, obstructive jaundice, or hypothyroidism.
6. Severe infectious diseases.

GDF-15 measurement: GDF-15 levels were measured using a double-antibody one-step sandwich enzyme-linked immunosorbent assay (ELISA).

### 2.3. Statistical Methods

Statistical analyses were performed using SPSS Version 26.0. Continuous variables with a normal distribution were expressed as mean ± standard deviation (SD). Comparisons between two groups were performed using the independent samples *t*-test; for comparisons involving more than two groups, one-way analysis of variance (ANOVA) was used. Nonnormally distributed data were presented as medians with interquartile ranges (IQRs), and differences between groups were analyzed using nonparametric tests. Categorical variables were presented as counts (percentages) and compared using the chi-square (*χ*^2^) test. Logistic regression models were constructed and evaluated using R software.

## 3. Results

There were no significant differences in age, gender, or BMI between the NDR and DR groups (*p* > 0.05), indicating comparability between the two groups. However, significant differences were observed in several variables, including COD, HB, ALB, UREA, DvT, LDH, HBTH, CO_2_, 2h-CP, and ApoA-1 (*p* < 0.05). The distribution patterns of these variables between the two groups are summarized in Table 1 (see Supporting Information 6: Table S1).

The DR group exhibited significantly lower levels of HB, ALB, CO_2_, and 2h-CP compared to the NDR group, whereas levels of DvT, UREA, HDL-C, ApoA-1, and GDF-15 were significantly higher. To identify predictive factors for DR, a logistic regression model was developed. Variable selection was performed using a stepwise regression approach. The dataset was randomly divided into a training set and a test set in an 8:2 ratio. Model performance was assessed using accuracy, sensitivity, specificity, F1-score, and the area under the receiver operating characteristic (ROC) curve (AUC) and compared with a model that included only GDF-15 as a predictor. Ultimately, six variables—ALB, ApoA-1, CO_2_, DvT, 2h-CP, and GDF-15—were incorporated into the final logistic regression model. The evaluation metrics of this model are summarized in Table 2.

**Figure 1:**
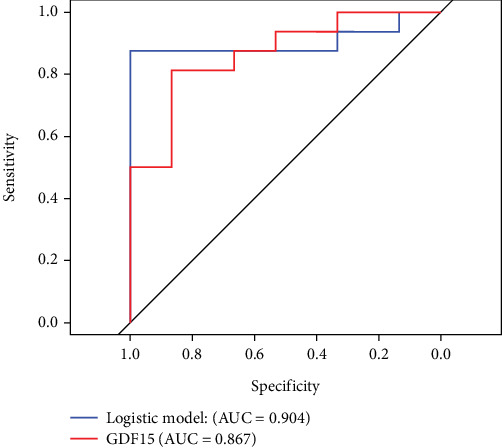
Model ROC curve.

The comprehensive indicators demonstrate that the constructed logistic regression model outperforms the model that uses only GDF-15 for prediction. The ROC curves comparing the performance of these two models are shown in Figure 1.

To further evaluate the overall performance of the model, we plotted the calibration curve (see Supporting Information 1: Figure S1) and the decision curve (see Supporting Information 2: Figure S2) for the logistic regression model. The results indicated that the model demonstrates strong performance.

The logistic regression formula is as follows:

 $$\begin{array}{c} \text{LogitP}=1.60365-0.10135\times \text{ALB}-0.17334\times {\text{CO}}_{2}+0.83592\times \text{ApoA}-1+0.71851\times \text{GDF}‐15+0.51620\times \text{DvT}-0.13672\times 2\text{h}‐\text{CP} \end{array}$$

To further improve the interpretability and visualization of the model, a nomogram was constructed (see Supporting Information 3: Figure S3), providing an intuitive representation of the impact of different factors on DR.

Since the regression coefficient *β*_1_ reflects the change in LogitP corresponding to a one-unit change in the independent variable *X*_1_, the odds ratios (ORs) for the relevant risk factors associated with DR are presented in Supporting Information 4: Figure S4, along with detailed information in Table 3.

The results indicate that CO_2_ (OR = 0.84, 95% CI: [0.72, 0.99]) and 2h-CP (OR = 0.87, 95% CI: [0.76, 0.99]) are statically significant protective factors for DR (*p* < 0.05). In contrast, ApoA-1 (OR = 2.31, 95% CI: [1.24, 4.30]) and GDF-15 (OR = 2.05, 95% CI: [1.27, 3.32]) are statistically significant risk factors for DR (*p* < 0.05).

To further explore potential correlations among the influencing factors, a correlation heatmap was generated (see Supporting Information 5: Figure S5). The results showed no significant correlations between the variables.

**Figure 2:**
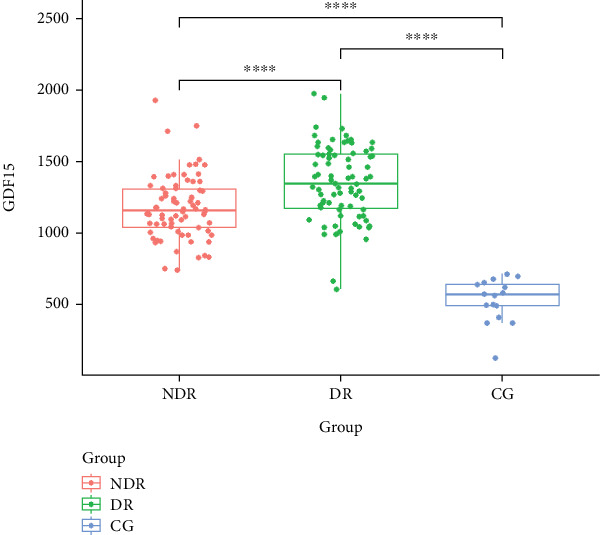
Serum GDF-15 levels in different groups.

To assess differences in serum GDF-15 levels across various stages of diabetes, 17 healthy controls (CG group) were included. ANOVA was conducted to evaluate significant differences among the three groups (*p* < 0.05). The results are presented in Figure 2, with detailed findings provided in Table 4.

Significant differences in serum GDF-15 levels were observed at various stages of DR progression. The DR group exhibited the highest level (1351.67 ± 261.92 pg/mL), followed by the NDR group (1179.43 ± 220.57 pg/mL). The CG group has the lowest level (533.92 ± 149.61 pg/mL). The findings tentatively suggest that serum GDF-15 increases with the progression of Type 2 diabetes.

In summary, a logistic regression model was developed and evaluated, showing strong performance. Our analysis of GDF-15 distribution across different stages of Type 2 diabetes indicated that GDF-15 levels increase with both the occurrence and progression of the disease.

## 4. Discussion

DR is a prevalent and severe chronic complication of diabetes that significantly impairs patients' quality of life and can ultimately lead to complete vision loss. As such, the development of effective strategies for its early prevention and treatment is of critical clinical importance.

DR is typically classified into two stages. Nonproliferative diabetic retinopathy (NPDR) represents the early stage, characterized by pathological changes such as basement membrane thickening, disruption of tight junctions between pericytes and endothelial cells, and breakdown of the blood–retinal barrier (BRB). Other features of NPDR include pericyte loss and endothelial cell dysfunction, which increase the fragility of retinal capillaries. These changes can result in microaneurysms, retinal hemorrhages, cotton wool spots, and capillary ischemia. As the disease progresses, ischemia induces the release of several factors, including vascular endothelial growth factor (VEGF) [18], transforming growth factor-*β* (TGF-*β*), basic fibroblast growth factor (bFGF), hepatocyte growth factor (HGF), stromal cell–derived factor-1 (SDF-1), connective tissue growth factor (CTGF), erythropoietin (EPO), and neurotrophic factor (NTS). These contribute to retinal neovascularization and progression to PDR [19], characterized by fragile new vessels that may lead to retinal and vitreous hemorrhages, tractional retinal detachment, and potential loss of vision.

GDF-15, a member of the TGF-*β* superfamily [20], plays a significant role in cellular immunity and proliferation [21]. It is regarded as a stress response cytokine [22], with physiological functions similar to those of TGF-*β*, including roles in cell growth, differentiation, and tissue inflammation. During cellular or tissue damage, endothelial cells and macrophages secrete GDF-15 [23]. Previous research has demonstrated that GDF-15 affects metabolism and insulin sensitivity by activating GFRAL, suggesting its involvement in metabolic disorders, oxidative stress responses, and inflammation. Recent studies have identified GDF-15 as a key player in neurovascular regulation associated with DR. It has been shown to reduce age-related inflammation, thereby helping to maintain glucose homeostasis and insulin sensitivity in both humans and mice [24]. As an adipokine, GDF-15 plays a crucial role in regulating body weight and food intake [25]. Its association with obesity, insulin resistance, and T2DM is well-established [26], with GDF-15 levels positively correlated with impaired fasting glucose (IFG) and newly diagnosed diabetes (NDD) [20]. Consequently, GDF-15 is considered a valuable biomarker for both obesity and T2DM [27].

GDF-15 activates GFRAL, influencing metabolism and insulin sensitivity, and may be involved in the development of metabolic disorders, oxidative stress, and inflammation. Recently, the role of adipocytokines in DR has also been highlighted. Early studies indicated that elevated GDF-15 levels are associated with retinal inflammation and damage. For instance, GDF-15 levels in the retina significantly increase following optic nerve damage [28]. Additionally, elevated GDF-15 has been reported to be considerably higher in the inflammatory vitreous of PDR [15]. GDF-15 is positively correlated with the severity of DR [16, 17]. This study did not find a difference between GDF-15 and other factors, such as DN, peripheral neuropathy, neck blood vessels, arteriovenous plaques in the lower limbs, BMI, systolic blood pressure, albumin-to-creatinine ratio, creatinine, and liver enzymes. It was also negatively correlated with glomerular filtration rate [17]. In our study, no significant correlation was found between GDF-15 and other factors such as DN, peripheral neuropathy, neck blood vessels, arteriovenous plaques in the lower limbs, BMI, or systolic blood pressure. However, GDF-15 was positively correlated with DR, suggesting that it may serve as a risk factor for DR. Collectively, both animal and human studies indicate that GDF-15 levels are significantly elevated in patients with DR. The pathogenesis may involve mechanisms such as endothelial cell proliferation, endothelial cell adhesion, retinal neovascularization, and extracellular matrix fibrosis. Despite this, some studies suggest that GDF-15 may also be a biomarker for DN [29]. Given these varied findings, our exploration of the relationship between diabetes, its complications, and GDF-15, including comparisons with healthy controls, highlights that while GDF-15 is associated with DR, it does not show significant correlations with other diabetic complications in our cohort.

HDL and ApoA-1 are thought to influence glucagon regulation [30]. Studies have shown that ApoA-1 positively promotes glucose uptake by skeletal muscle and myocardial cells [31]. However, some evidence suggests that it may be associated with the occurrence of diabetic macular edema (DMO) [32]. Conversely, other studies report a negative correlation between the severity of DR and ApoA-1 levels [33]. In our study, ApoA-1 was identified as a risk factor for DR, showing a positive correlation with its presence.

Previous research has demonstrated that CO_2_ laser therapy can enhance wound healing in individuals with diabetes. In our study, CO_2_ was identified as a protective factor for DR. C-peptide, a biologically active peptide hormone, has been linked to diabetic microvascular complications in T2DM [34]. Our results indicate that 2-h postprandial C-peptide is significantly associated with DR and functions as a protective factor against its development. This study has certain limitations. First, it is specific to the Handan region, which may limit the generalizability of the findings. Second, the relatively small sample size may introduce bias. Despite these limitations, our findings provide valuable insights into the role of GDF-15 and its association with DR in patients with T2DM, highlighting GDF-15 as a potential risk factor for DR.

In summary, GDF-15 shows promise as a biomarker for the early detection of DR. This study uniquely included a healthy control group and provided a comprehensive analysis of GDF-15 regarding macrovascular diseases, such as DN, coronary heart disease, and cerebral infarction. Our results suggest that elevated levels of GDF-15 and ApoA-1 are risk factors for DR, while CO_2_ and 2-h postprandial C-peptide act as protective factors. GDF-15 may offer a novel approach for the early diagnosis of DR; however, further large-scale studies are needed to clarify the underlying mechanisms linking GDF-15 to DR.

## 5. Conclusions

In individuals with T2DR, the expression level of GDF-15 was significantly upregulated. GDF-15 is strongly associated with the severity of DR and serves as an independent risk factor for its development.

A statistically significant correlation was also observed between ApoA-1 and DR, suggesting that changes in ApoA-1 levels may represent a potential risk factor for DR. In contrast, both CO_2_ levels and 2-h postprandial C-peptide levels were significantly associated with DR, acting as protective factors against its occurrence.

## Figures and Tables

**Table 1:** Distribution of patient data across different groups.

	**NDR (** **N** = 74**)**	**DR (** **N** = 79**)**	**p**
HB^a^	147.81 ± 17.29	141.08 ± 19.47	0.025
DvT^a^	0.22 ± 0.12	0.28 ± 0.22	0.029
ALB^a^	43.14 ± 3.52	41.53 ± 4.04	0.010
UREA^a^	5.60 ± 1.95	6.28 ± 1.79	0.026
CO_2_^a^	26.44 ± 2.72	25.47 ± 2.55	0.025
HDL-C^a^	0.93 ± 0.23	1.03 ± 0.30	0.022
ApoA-1^a^	1.34 ± 0.19	1.45 ± 0.20	0.002
2h-CP^a^	5.36 ± 3.73	3.91 ± 2.80	0.007
GDF-15^a^	1129.43 ± 297.89	1349.01 ± 306.69	< 0.001

Abbreviations: 2h-CP, 2-h postprandial C-peptide; ALB, albumin; ApoA-1, apolipoprotein A1; CO_2_, total carbon dioxide; DvT, d-dimer; HB, hemoglobin; HDL-C, high-density lipoprotein cholesterol; GDF-15, growth differentiation factor-15; UREA, urea.

^a^Variables with significant differences between the two sets of data.

**Table 2:** Model evaluation results.

	**Accuracy**	**Sensitivity**	**Specificity**	**F1**	**AUC**
Comprehensive model	0.936	1.000	0.875	0.938	0.904
GDF-15	0.839	0.867	0.813	0.839	0.867

**Table 3:** Multivariate logistic regression analysis of factors affecting the occurrence of DR.

	**B**	**Wald**	**OR (95% CI)**	**p**
ALB	−0.101	2.765	0.904 (0.799~1.016)	0.096
CO_2_	−0.173	4.481	0.841 (0.711~0.983)	0.034
ApoA-1	0.836	6.898	2.307 (1.277~4.49)	0.009
GDF-15	0.719	8.530	2.051 (1.299~3.443)	0.003
DvT	0.516	2.684	1.676 (0.931~3.242)	0.101
2h-CP	−0.137	4.110	0.872 (0.759~0.991)	0.043

**Table 4:** GDF-15 content in different groups.

	**NDR**	**DR**	**CG**	**p**
GDF-15	1179.43 ± 220.57	1351.67 ± 261.92	533.92 ± 149.61	< 0.001

## Data Availability

The datasets generated during and analyzed during the current study are available from the corresponding authors on reasonable request.

## Supporting Information

Supporting Information 1 Figure S1: Model calibration curve. This figure shows the calibration performance of the logistic regression model for predicting diabetic retinopathy.

Supporting Information 2 Figure S2: Model decision curve. This figure presents the decision curve analysis demonstrating the clinical net benefit of the prediction model.

Supporting Information 3 Figure S3: Nomogram model for DR occurrence. This nomogram provides a visual representation of the logistic regression model for individualized prediction of diabetic retinopathy risk.

Supporting Information 4 Figure S4: Odds ratio (OR) values of relevant influencing factors. This figure displays the ORs and 95% CIs for each variable included in the multivariate logistic regression model.

Supporting Information 5 Figure S5: Correlation heatmap between different variables. This heatmap illustrates the correlations among the included clinical and biochemical parameters.

Supporting Information 6 Table S1: Baseline clinical and biochemical characteristics of patients in the NDR and DR groups. This table provides the detailed distribution of patient demographic and laboratory data used for statistical analysis.

Supporting Information 7 Informed consent document.

Supporting Information 8 Ethical review document.
