# Supplementary material for: Study on the Correlation Between GDF-15 Levels and a Diagnostic Model for Diabetic Retinopathy
Source: J Diabetes Res. 2025 Sep 18;2025:6959604. doi: 10.1155/jdr/6959604 (PMC12463507; doi:10.1155/jdr/6959604)
Supplement: Supporting Information 1 — Figure S1: Model calibration curve. This figure shows the calibration performance of the logistic regression model for predicting diabetic retinopathy. [file 6959604.f1.docx]

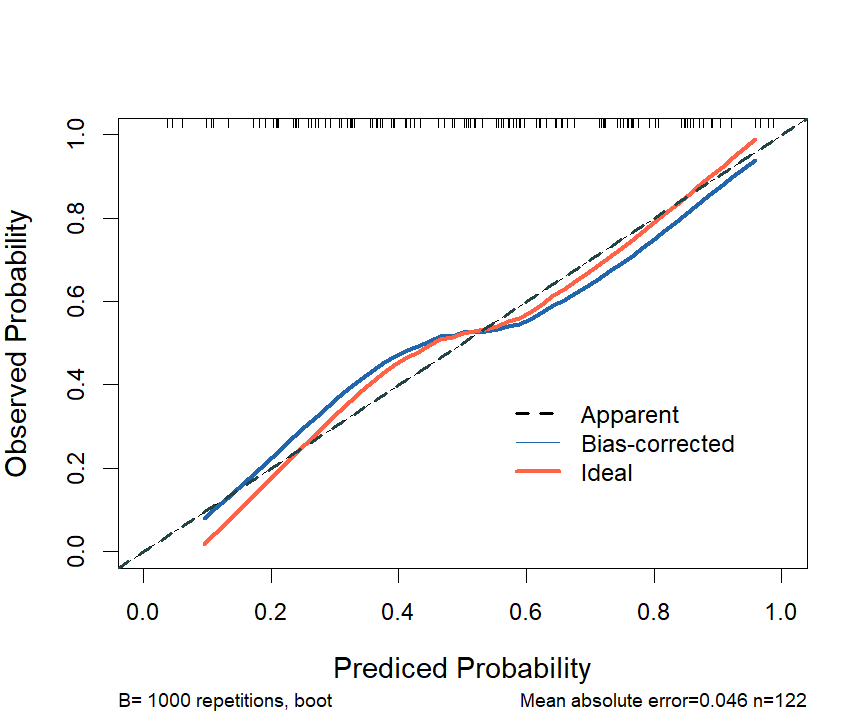


**Figure S1.** **Model calibration curve.** This figure shows the calibration performance of the logistic regression model for predicting diabetic retinopathy.
