# Supplementary material for: Study on the Correlation Between GDF-15 Levels and a Diagnostic Model for Diabetic Retinopathy
Source: J Diabetes Res. 2025 Sep 18;2025:6959604. doi: 10.1155/jdr/6959604 (PMC12463507; doi:10.1155/jdr/6959604)
Supplement: Supporting Information 2 — Figure S2: Model decision curve. This figure presents the decision curve analysis demonstrating the clinical net benefit of the prediction model. [file 6959604.f2.docx]

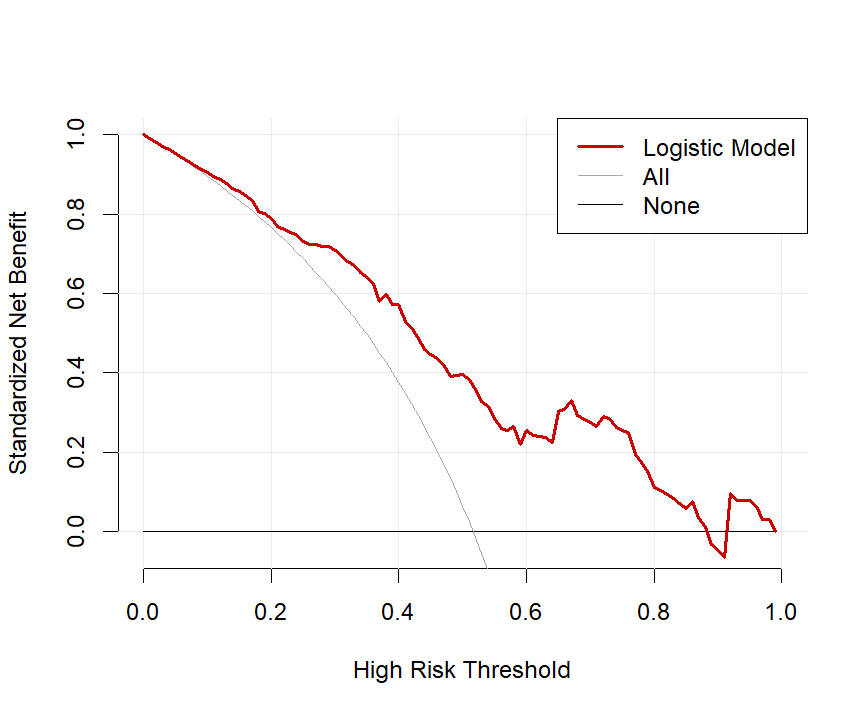


**Figure S2.** **Model decision curve.** This figure presents the decision curve analysis demonstrating the clinical net benefit of the prediction model.
