# Supplementary material for: Study on the Correlation Between GDF-15 Levels and a Diagnostic Model for Diabetic Retinopathy
Source: J Diabetes Res. 2025 Sep 18;2025:6959604. doi: 10.1155/jdr/6959604 (PMC12463507; doi:10.1155/jdr/6959604)
Supplement: Supporting Information 3 — Figure S3: Nomogram model for DR occurrence. This nomogram provides a visual representation of the logistic regression model for individualized prediction of diabetic retinopathy risk. [file 6959604.f3.docx]

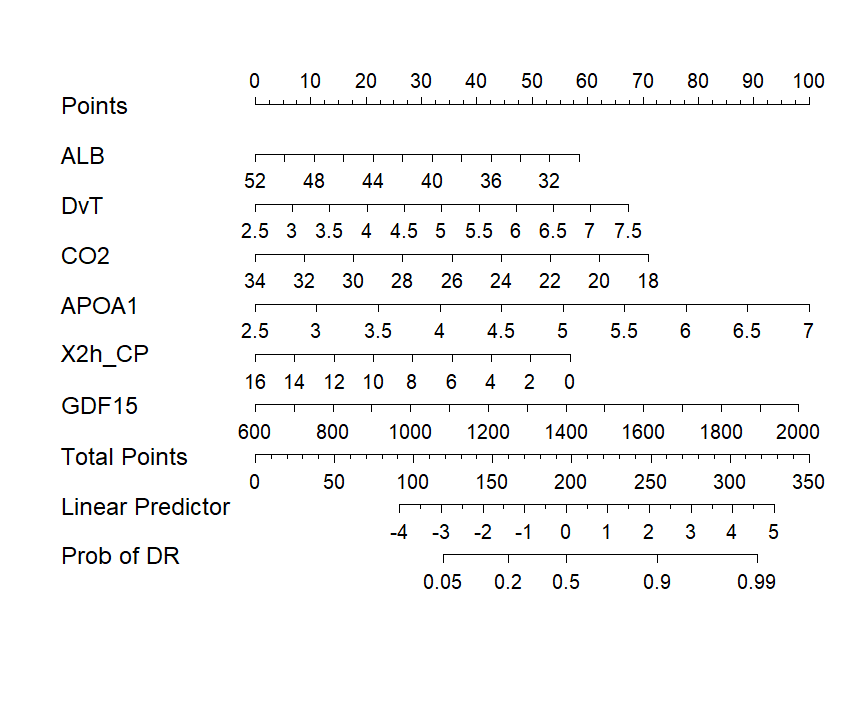


**Figure S3. Nomogram model for DR occurrence.** This nomogram provides a visual representation of the logistic regression model for individualized prediction of diabetic retinopathy risk.
