# Supplementary material for: Study on the Correlation Between GDF-15 Levels and a Diagnostic Model for Diabetic Retinopathy
Source: J Diabetes Res. 2025 Sep 18;2025:6959604. doi: 10.1155/jdr/6959604 (PMC12463507; doi:10.1155/jdr/6959604)
Supplement: Supporting Information 4 — Figure S4: Odds ratio (OR) values of relevant influencing factors. This figure displays the ORs and 95% CIs for each variable included in the multivariate logistic regression model. [file 6959604.f4.docx]

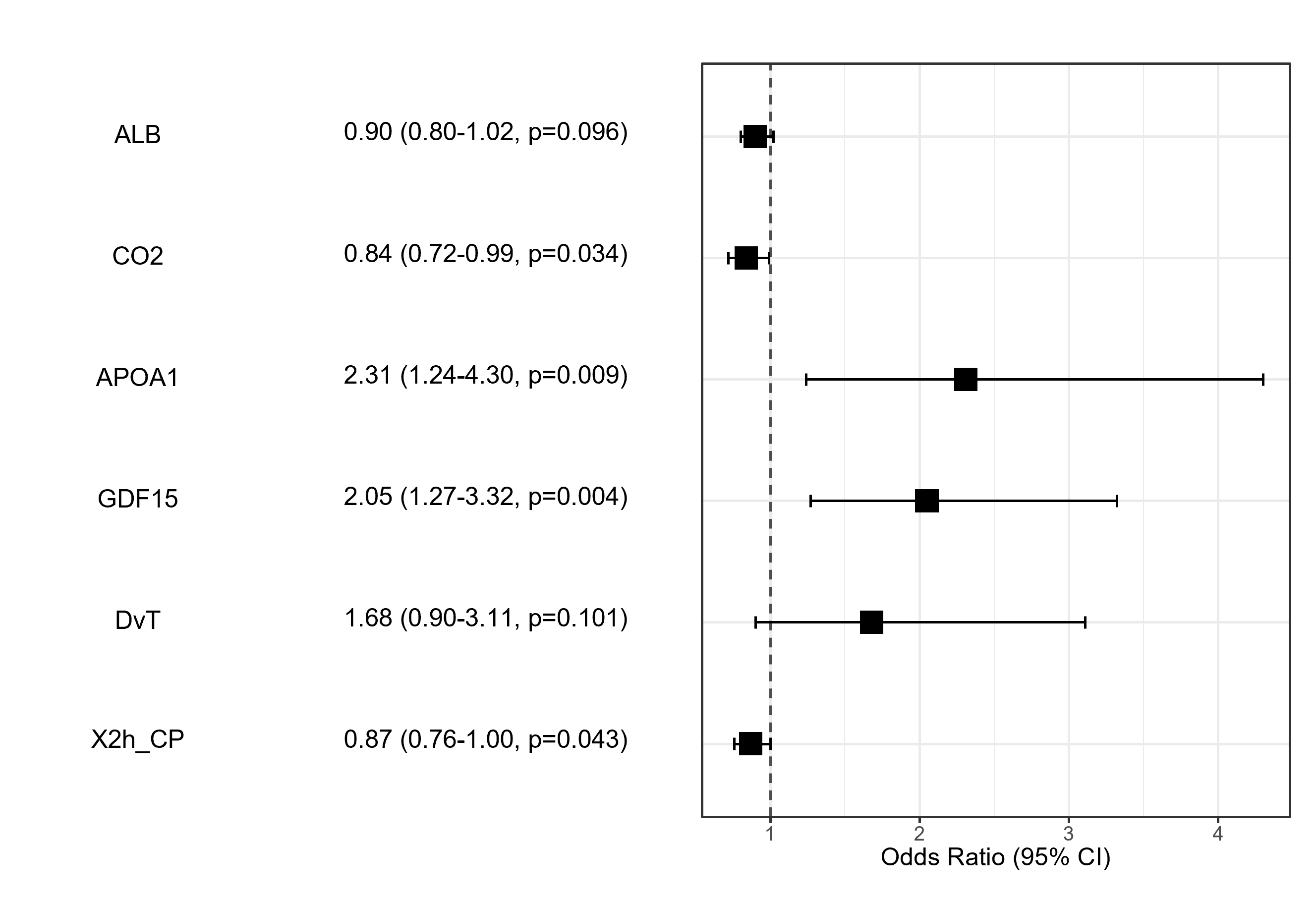


**Figure S4.** **OR values of relevant influencing factors.** This figure displays the ORs and 95% CIs for each variable included in the multivariate logistic regression model.
