# Supplementary material for: Study on the Correlation Between GDF-15 Levels and a Diagnostic Model for Diabetic Retinopathy
Source: J Diabetes Res. 2025 Sep 18;2025:6959604. doi: 10.1155/jdr/6959604 (PMC12463507; doi:10.1155/jdr/6959604)
Supplement: Supporting Information 5 — Figure S5: Correlation heatmap between different variables. This heatmap illustrates the correlations among the included clinical and biochemical parameters. [file 6959604.f5.docx]

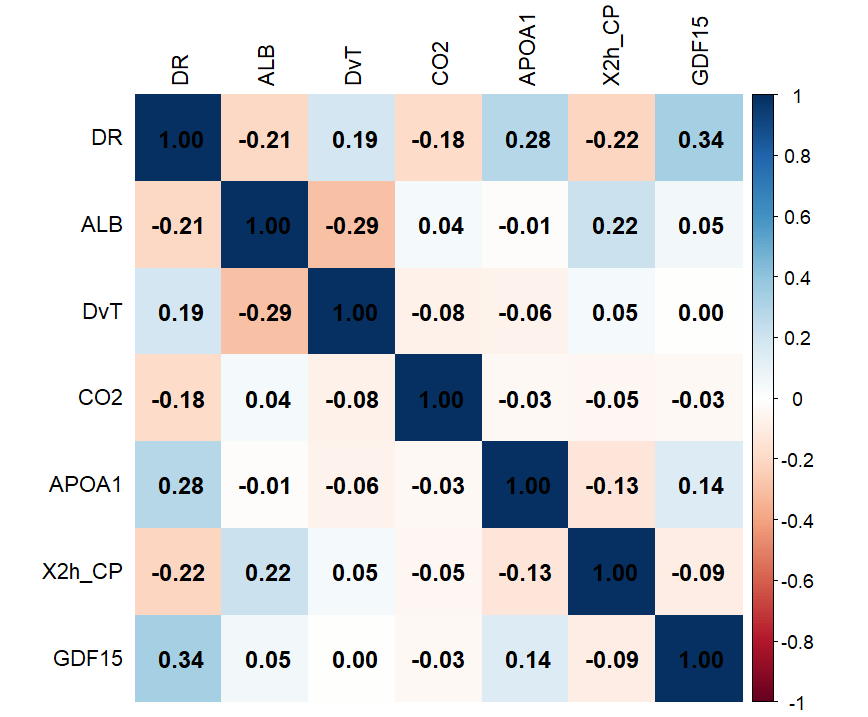


**Figure S5. Correlation heatmap between different variables.** This heatmap illustrates the correlations among the included clinical and biochemical parameters.
