# Supplementary material for: Study on the Correlation Between GDF-15 Levels and a Diagnostic Model for Diabetic Retinopathy
Source: J Diabetes Res. 2025 Sep 18;2025:6959604. doi: 10.1155/jdr/6959604 (PMC12463507; doi:10.1155/jdr/6959604)
Supplement: Supporting Information 6 — Table S1: Baseline clinical and biochemical characteristics of patients in the NDR and DR groups. This table provides the detailed distribution of patient demographic and laboratory data used for statistical analysis. [file 6959604.f6.docx]

**Table S1.** Distribution trend of patient data in different groups

|  | NDR (N=74) | DR (N=79) | *P* |
| --- | --- | --- | --- |
| Gender |  |  | 0.479 |
| Female | 24 (32.4%) | 31 (39.2%) |  |
| Male | 50 (67.6%) | 48 (60.8%) |  |
| Age（year） | 54.04 ± 13.16 | 56.51 ± 10.18 | 0.199 |
| Weight(kg) | 75.42 ± 14.95 | 72.17 ± 14.77 | 0.179 |
| BMI(kg/m2) | 26.30 ± 3.84 | 25.83 ± 4.37 | 0.481 |
| SBP(mmHg) | 135.08 ± 14.94 | 137.54 ± 19.75 | 0.384 |
| DBP(mmHg) | 89.01 ± 9.13 | 88.46 ± 11.60 | 0.741 |
| Smoke |  |  | 0.115 |
| No | 53 (71.6%) | 66 (83.5%) |  |
| Yes | 21 (28.4%) | 13 (16.5%) |  |
| Drink |  |  | 1.000 |
| No | 51 (68.9%) | 55 (69.6%) |  |
| Yes | 23 (31.1%) | 24 (30.4%) |  |
| FH |  |  | 0.651 |
| No | 43 (58.1%) | 42 (53.2%) |  |
| Yes | 31 (41.9%) | 37 (46.8%) |  |
| HBH |  |  | 0.759 |
| No | 44 (59.5%) | 44 (55.7%) |  |
| Yes | 30 (40.5%) | 35 (44.3%) |  |
| COD* | 99.11 ± 89.01 | 134.73 ± 82.78 | 0.011 |
| intervention |  |  | 0.021 |
| No | 15 (20.3%) | 5 (6.3%) |  |
| Yes | 59 (79.7%) | 74 (93.7%) |  |
| PN |  |  | 0.664 |
| No | 6 (8.1%) | 4 (5.1%) |  |
| Yes | 68 (91.9%) | 75 (94.9%) |  |
| NV |  |  | 0.538 |
| No | 24 (32.4%) | 21 (26.6%) |  |
| Yes | 50 (67.6%) | 58 (73.4%) |  |
| BVP |  |  | 0.529 |
| No | 27 (36.5%) | 24 (30.4%) |  |
| Yes | 47 (63.5%) | 55 (69.6%) |  |
| LEVD |  |  | 0.388 |
| No | 5 (6.8%) | 2 (2.5%) |  |
| Yes | 69 (93.2%) | 77 (97.5%) |  |
| WC(cm) | 94.14 ± 11.37 | 92.79 ± 11.88 | 0.477 |
| HC(cm) | 99.11 ± 7.04 | 98.28 ± 15.20 | 0.663 |
| WHR | 0.94±0.07 | 0.96±0.18 | 0.630 |
| HOMA.IR | 3.64 ± 4.29 | 4.08 ± 5.50 | 0.579 |
| TyG | 2.86 ± 0.75 | 2.98 ± 0.79 | 0.366 |
| WBC(10^9/L) | 6.53 ± 1.77 | 6.51 ± 2.07 | 0.953 |
| RBC(10^12/L) | 4.95 ± 0.60 | 4.80 ± 0.68 | 0.156 |
| HB*(g/l) | 147.81 ± 17.29 | 141.08 ± 19.47 | 0.025 |
| PLT(10^9/L) | 238.80 ± 65.01 | 232.16 ± 61.88 | 0.519 |
| FIB(g/l) | 3.01 ± 0.73 | 3.23 ± 1.56 | 0.252 |
| DvT*(g/l) | 0.22 ± 0.12 | 0.28 ± 0.22 | 0.029 |
| ALT(U/L) | 27.00 ± 15.32 | 24.28 ± 16.88 | 0.299 |
| ASTU/L) | 21.14 ± 8.55 | 21.22 ± 10.64 | 0.959 |
| ALT/AST | 1.25±0.36 | 1.11±0.36 | 0.013 |
| TP(g/l) | 69.57 ± 6.27 | 68.16 ± 6.04 | 0.158 |
| ALB*(g/l) | 43.14 ± 3.52 | 41.53 ± 4.04 | 0.010 |
| GLO(g/l) | 26.43 ± 4.15 | 26.63 ± 3.58 | 0.748 |
| A/G | 1.67±0.25 | 1.59±0.23 | 0.046 |
| TBIL(umol/l) | 14.07±5.29 | 12.62±4.34 | 0.065 |
| DBIL(umol/l) | 2.62 ± 1.13 | 2.29 ± 0.98 | 0.055 |
| IBIL(umol/l) | 11.45 ± 4.39 | 10.34 ± 3.61 | 0.086 |
| CHE(U/L) | 10006.04 ± 2222.73 | 9908.42 ± 2565.77 | 0.802 |
| ALP(U/L) | 85.86 ± 28.33 | 84.32 ± 29.28 | 0.740 |
| GGT(U/L) | 33.82 ± 24.10 | 36.51 ± 38.07 | 0.601 |
| PA(g/l) | 0.28 ± 0.06 | 0.28 ± 0.07 | 0.967 |
| UREA*(mmol/l) | 5.60 ± 1.95 | 6.28 ± 1.79 | 0.026 |
| CREA(umol/l) | 64.19 ± 20.62 | 68.01 ± 26.59 | 0.321 |
| eGFR(ml/min) | 102.92 ± 20.24 | 97.11 ± 21.85 | 0.091 |
| UA(umol/l) | 328.21 ± 111.86 | 300.09 ± 87.76 | 0.087 |
| GLU(mmol/l) | 11.93 ± 4.48 | 12.46 ± 5.15 | 0.500 |
| Ca(mmol/l) | 2.33 ± 0.10 | 2.30 ± 0.10 | 0.062 |
| P(mmol/l) | 1.15 ± 0.18 | 1.14 ± 0.17 | 0.699 |
| Na(mmol/l) | 137.16 ± 3.10 | 136.86 ± 3.15 | 0.542 |
| K(mmol/l) | 4.19 ± 0.39 | 4.16 ± 0.38 | 0.539 |
| CL(mmol/l) | 102.06 ± 3.27 | 102.35 ± 3.31 | 0.594 |
| CO_2_*(mmol/l) | 26.44 ± 2.72 | 25.47 ± 2.55 | 0.025 |
| TCH(mmol/l) | 4.55 ± 1.00 | 4.89 ± 1.41 | 0.086 |
| TG(mmol/l) | 1.84±1.14 | 2.19±2.02 | 0.189 |
| HDL-C*(mmol/l) | 0.93 ± 0.23 | 1.03 ± 0.30 | 0.022 |
| LDH-C(mmol/l) | 2.74 ± 0.77 | 2.88 ± 0.98 | 0.320 |
| APOA-1*(g/l) | 1.34 ± 0.19 | 1.45 ± 0.20 | 0.002 |
| APOB(g/l) | 0.89 ± 0.24 | 0.93 ± 0.29 | 0.390 |
| APOa(g/l) | 0.18 ± 0.25 | 1.00 ± 7.09 | 0.308 |
| ADA(U/L) | 13.41 ± 5.36 | 14.83 ± 5.59 | 0.112 |
| HbA1c(%) | 9.59 ± 1.99 | 9.81 ± 2.09 | 0.512 |
| FPG(mmol/l) | 7.16 ± 1.90 | 7.50 ± 2.03 | 0.276 |
| 2h.PG(mmol/l) | 10.56 ± 3.98 | 10.95 ± 3.41 | 0.523 |
| FCP(ng/ml) | 2.13 ± 1.64 | 1.90 ± 1.41 | 0.337 |
| FINS(uIU/ml) | 11.31 ± 13.65 | 11.47 ± 12.77 | 0.939 |
| 2h-CP*(ng/ml) | 5.36 ± 3.73 | 3.91 ± 2.80 | 0.007 |
| 2h.ins(uIU/ml) | 37.44 ± 31.16 | 37.66 ± 36.75 | 0.969 |
| CK(U/L) | 85.35 ± 62.64 | 87.18 ± 58.35 | 0.852 |
| CK-MB(U/L) | 10.53 ± 2.76 | 11.44 ± 3.77 | 0.087 |
| LDH*(U/L) | 160.64 ± 34.77 | 172.22 ± 35.86 | 0.045 |
| HBTH*(U/L) | 103.45 ± 23.74 | 111.42 ± 22.24 | 0.034 |
| Mb(ng/ml) | 30.14 ± 28.47 | 30.67 ± 17.74 | 0.890 |
| GDF-15*(pg/ml) | 1129.43 ± 297.89 | 1349.01 ± 306.69 | <.001 |

*Variables with significant differences between the two sets of data

BM: body mass index; SBP: systolic blood pressure; DBP: diastolic blood pressure; FH: Family history; COD: Course of disease; HBH: History of hypertension; PN: Peripheral neuropathy; NV: Vascular disease of the neck; BVP: Venous plaques in both lower extremities; LEVD: Lower extremity vascular disease; WC: waist circumference; HC: hip circumference; WHR: Waist-hip ratio; HOMA-IR: Insulin resistance index; TyG: Triglyceride Glucose Index; WBC: White blood cell count; RBC: Red blood cell count; PLT：Platelet count; FIB: Fibinogen； ALT：alanine transaminase; AST：aspartate aminotransferase；AST/ALT: Albumin/Globulin; TP: Total protein；GLO：Globulin：A/G：Albumin/Globulin; TBIL：Indirect bilirubin; DBIL: direct bilirubin; IBIL: Indirect Bilirubin; CHE: Cholinesterase；ALPAl：kaline phosphatase; GGT: Glutamyl transpeptidase; PA: Prealbumin; CREA: creatinine; eGFR: estimated glomerular filtration rate; UA: Uric acid; GLU: Glucose; Ca: Calcium; P: Phosphorus; Na Sodium; K:Potassium;CL:Chloride; TCH: serum total cholesterol；TG: Triglyceride； LDL-C: low density lipoprotein cholesterol； ApoB: Apolipoprotein B; Apoa: Lipoprotein a; ADA: Adenosine deaminase; HbA1c: haemoglobinA1c; FPG: fasting plasma glucose; 2H-PG:2-hour postprandial blood glucose; FI: Fasting insulin; 2h-INS:2-hour postprandial insulin; FC-P: Fasting C-peptide; CK: Creatine kinase; CK-MB: Creatine kinase isoenzyme; LDH: Lactate dehydrogenase; HBDH Hydroxybutyrate dehydrogenase; hs-CRP: High-sensitivity C-reactive protein; MB: Myoglobin;
