# Supplementary material for: Study on the Correlation Between GDF-15 Levels and a Diagnostic Model for Diabetic Retinopathy
Source: J Diabetes Res. 2025 Sep 18;2025:6959604. doi: 10.1155/jdr/6959604 (PMC12463507; doi:10.1155/jdr/6959604)
Supplement: Supporting Information 7 — Informed consent document. [file 6959604.f7.pdf]

Handan Central Hospital
Informed consent of scientific research project

Project name:	Study on the Correlation Between GDF-15 Levels and a Diagnostic Model for Diabetic Retinopathy
Version number and date:	1.0 June 25, 2024
Undertaking department	Endocrinology department 1
Main researchers	Mingming Zhu and Xuenan Lian

Dear patients:

We invite you to participate in a clinical trial. This informed consent form provides you with some information to help you decide whether to participate in this clinical trial. Please take some time to read the following contents carefully. If you have unclear questions or terms, you can discuss them with the doctors concerned.

Your participation in this study is voluntary. This study has been reviewed by the Scientific Research Ethics Committee of Handan Central Hospital.

1. Research purposes:

The purpose of this study is to explore the correlation between GDF-15 level and the risk of diabetic retinopathy in patients with T2DM in Handan, China.

2. the research process:

Research content: Diabetic retinopathy (DR) is a common microvascular complication of diabetes, which affects about one third of diabetic patients and seriously threatens their eyesight. In recent years, the role of inflammatory biomarkers and cytokines in DR has gradually attracted attention. We selected patients with type 2 diabetes mellitus (T2DM) in Handan Central Hospital in 2024 as the research object, and divided them into diabetic retinopathy (DR) group with 74 cases and diabetic retinopathy (DR) group with 79 cases. Stepwise regression method was used to screen variables and build a Logistic regression clinical prediction model. In order to further explore the relationship between the occurrence of DR and GDF15, 17 healthy people (CG) were included.

Inspection operation and use: only 5ml of arm venous blood is needed for the test, which will not cause serious harm to your body. After the blood sample is centrifuged by a centrifuge, it is quickly put into a freezing tube, and then the freezing tube is stored at -80℃ in our hospital. The levels of GDF-15 among the three groups were detected by double antibody one-step sandwich enzyme-linked immunosorbent assay (ELISA). In addition to clinical testing purposes, we may conduct more in-depth scientific research on your blood samples and use the results for publishing papers, but we will not use them for commercial purposes.

3. Risks and discomfort of participating in the research:

Risk and discomfort: The risk of blood drawing includes temporary discomfort and/or cyanosis. Although unlikely, infection, bleeding, coagulation or syncope may occur.

4. Benefits from participating in the research:

If you agree to participate in this research, you will not benefit. We hope that the information obtained from your participation in this study will be instructive to patients with the same condition as you in the future.

5. Related expenses for participating in the research:

You don't have to pay any fees.

6. compensation:

You will not be compensated for donating samples.

7. Right to refuse to participate in or withdraw from the research:

You can choose not to participate in this study, or you have the right to withdraw at any stage of the study without any reason, and your medical treatment and rights will not be affected. Once you decide to participate in this study, please sign this informed consent form to show your consent. Before entering the study, the doctor will screen you to confirm whether you are a suitable candidate.

If you choose to participate in this research, we hope that you can persist in completing the whole research process.

If you need other treatment, or if you fail to follow the research plan, or if there are research-related injuries or for any other reason, the researcher may terminate your participation in this study. At that time, your sample will be destroyed, and you can withdraw at any time without any reason, and it will not affect your treatment.

8. Obligation to participate in research:

If you choose to participate in this project, you need to provide the researchers with the real situation about your medical history and current physical condition, and fill in relevant information; Tell the researcher about any discomfort you have experienced during this study; Tell the researchers whether they have participated in other studies recently or are currently participating in other studies. And other related matters that may affect the research results or your health.

9. Privacy and confidentiality:

During the research period, your personal data such as name and gender will be replaced by codes or numbers, and will be kept strictly confidential. Only the relevant doctors know your information, and your privacy will be well protected. The research results may be published in magazines, but no personal information will be disclosed.

If you agree to participate in this study, all of your medical information will be accessed by the appropriate personnel at the research and development unit that initiated this study, by relevant authorities, or by an independent ethics committee to check the appropriateness of the study's operation. By signing the informed consent form, you are agreeing to be accessed by these people.

10. How to get help in research:

You can keep abreast of the information and research progress related to this study. If you have any questions related to this study, please contact Mingming Zhu at 15233895369.

If you feel that your rights and interests have been damaged during the research, you can contact the Ethics Committee of Scientific Research of Handan Central Hospital at 0310-2112086.

Subject statement

I have carefully read the above contents, and have fully understood the purpose, content, methods and possible benefits and risks of participating in the clinical study. The doctor has clearly explained the relevant medical terms, and all the questions I asked have been answered easily. I understand that I can refuse to join the study or suspend and withdraw from the study at any time and under any circumstances, and my medical treatment and rights will not be affected.

My participation in this research is completely voluntary, and I have fully considered it. I have understood the benefits and possible risks brought by participating in this research, and obtained complete and true information related to this research. I fully understand and support this clinical research. Without any pressure and free choice, I volunteered to participate in this clinical study and cooperate with the research doctor to complete this clinical study.

I will get a signed and dated copy of the informed consent form.

患者（签名）：[illegible]

（或法定代理人（签名）：）__________ 与患者关系：

日期：[illegible]年[illegible]月[illegible]日

联系电话：[illegible]

Researcher's statement

I declare that I have explained the contents, steps, possible dangers and benefits of this study to the above-mentioned participants in detail, and have given full answers to any questions raised by the patients, and the patients have received satisfactory answers and expressed their understanding.

研究医生（签名）：[illegible]　　日期：2024年07月10日

联系电话：15233895369

Handan Central Hospital
Informed consent of scientific research project

Project name:	Study on the Correlation Between GDF-15 Levels and a Diagnostic Model for Diabetic Retinopathy
Version number and date:	1.0 June 25, 2024
Undertaking department	Endocrinology department 1
Main researchers	Mingming Zhu and Xuenan Lian

Dear patients:

We invite you to participate in a clinical trial. This informed consent form provides you with some information to help you decide whether to participate in this clinical trial. Please take some time to read the following contents carefully. If you have unclear questions or terms, you can discuss them with the doctors concerned.

Your participation in this study is voluntary. This study has been reviewed by the Scientific Research Ethics Committee of Handan Central Hospital.

1. Research purposes:

The purpose of this study is to explore the correlation between GDF-15 level and the risk of diabetic retinopathy in patients with T2DM in Handan, China.

2. the research process:

Research content: Diabetic retinopathy (DR) is a common microvascular complication of diabetes, which affects about one third of diabetic patients and seriously threatens their eyesight. In recent years, the role of inflammatory biomarkers and cytokines in DR has gradually attracted attention. We selected patients with type 2 diabetes mellitus (T2DM) in Handan Central Hospital in 2024 as the research object, and divided them into diabetic retinopathy (DR) group with 74 cases and diabetic retinopathy (DR) group with 79 cases. Stepwise regression method was used to screen variables and build a Logistic regression clinical prediction model. In order to further explore the relationship between the occurrence of DR and GDF15, 17 healthy people (CG) were included.

Inspection operation and use: only 5ml of arm venous blood is needed for the test, which will not cause serious harm to your body. After the blood sample is centrifuged by a centrifuge, it is quickly put into a freezing tube, and then the freezing tube is stored at -80℃ in our hospital. The levels of GDF-15 among the three groups were detected by double antibody one-step sandwich enzyme-linked immunosorbent assay (ELISA). In addition to clinical testing purposes, we may conduct more in-depth scientific research on your blood samples and use the results for publishing papers, but we will not use them for commercial purposes.

3. Risks and discomfort of participating in the research:

Risk and discomfort: The risk of blood drawing includes temporary discomfort and/or cyanosis. Although unlikely, infection, bleeding, coagulation or syncope may occur.

4. Benefits from participating in the research:

If you agree to participate in this research, you will not benefit. We hope that the information obtained from your participation in this study will be instructive to patients with the same condition as you in the future.

5. Related expenses for participating in the research:

You don't have to pay any fees.

6. compensation:

You will not be compensated for donating samples.

7. Right to refuse to participate in or withdraw from the research:

You can choose not to participate in this study, or you have the right to withdraw at any stage of the study without any reason, and your medical treatment and rights will not be affected. Once you decide to participate in this study, please sign this informed consent form to show your consent. Before entering the study, the doctor will screen you to confirm whether you are a suitable candidate.

If you choose to participate in this research, we hope that you can persist in completing the whole research process.

If you need other treatment, or if you fail to follow the research plan, or if there are research-related injuries or for any other reason, the researcher may terminate your participation in this study. At that time, your sample will be destroyed, and you can withdraw at any time without any reason, and it will not affect your treatment.

8. Obligation to participate in research:

If you choose to participate in this project, you need to provide the researchers with the real situation about your medical history and current physical condition, and fill in relevant information; Tell the researcher about any discomfort you have experienced during this study; Tell the researchers whether they have participated in other studies recently or are currently participating in other studies. And other related matters that may affect the research results or your health.

9. Privacy and confidentiality:

During the research period, your personal data such as name and gender will be replaced by codes or numbers, and will be kept strictly confidential. Only the relevant doctors know your information, and your privacy will be well protected. The research results may be published in magazines, but no personal information will be disclosed.

If you agree to participate in this study, all of your medical information will be accessed by the appropriate personnel at the research and development unit that initiated this study, by relevant authorities, or by an independent ethics committee to check the appropriateness of the study's operation. By signing the informed consent form, you are agreeing to be accessed by these people.

10. How to get help in research:

You can keep abreast of the information and research progress related to this study. If you have any questions related to this study, please contact Mingming Zhu at 15233895369.

If you feel that your rights and interests have been damaged during the research, you can contact the Ethics Committee of Scientific Research of Handan Central Hospital at 0310-2112086.

Subject statement

I have carefully read the above contents, and have fully understood the purpose, content, methods and possible benefits and risks of participating in the clinical study. The doctor has clearly explained the relevant medical terms, and all the questions I asked have been answered easily. I understand that I can refuse to join the study or suspend and withdraw from the study at any time and under any circumstances, and my medical treatment and rights will not be affected.

My participation in this research is completely voluntary, and I have fully considered it. I have understood the benefits and possible risks brought by participating in this research, and obtained complete and true information related to this research. I fully understand and support this clinical research. Without any pressure and free choice, I volunteered to participate in this clinical study and cooperate with the research doctor to complete this clinical study.

I will get a signed and dated copy of the informed consent form.

患者（签名）： [redacted]

（或法定代理人（签名）：） ____________ **与患者关系：**

日期： [redacted]年[redacted]月[redacted]日

联系电话： 18[redacted]0

Researcher's statement

I declare that I have explained the contents, steps, possible dangers and benefits of this study to the above-mentioned participants in detail, and have given full answers to any questions raised by the patients, and the patients have received satisfactory answers and expressed their understanding.

研究医生（签名）： [illegible] **日期：** 2024年07月22日

联系电话： 15233895369
