# Supplementary material for: Study on the Correlation Between GDF-15 Levels and a Diagnostic Model for Diabetic Retinopathy
Source: J Diabetes Res. 2025 Sep 18;2025:6959604. doi: 10.1155/jdr/6959604 (PMC12463507; doi:10.1155/jdr/6959604)
Supplement: Supporting Information 8 — Ethical review document. [file 6959604.f8.pdf]

Approval of ethical review

ApprovalNo.: (2024) Lun Shen PaperNo. (017)

Project name	Study on the Correlation Between GDF-15 Levels and a Diagnostic Model for Diabetic Retinopathy		
	Project source	Papers published	
	contract research organization		
Research unit	Handan Central Hospital		
Main researcher	Zhu Mingming	Belonging major	internal medicine
Review category	Initial review	Review mode	Rapid review of oral conference review port
Review date	2024. 10. 31	Review location	
	Presiding member	Zhang Hongfeng	
Review data	1、 Initial review application (scientific research project); 2、 Statement of economic interests of researchers;<br>3、 Clinical scientific research scheme; 4、 Exempt from the application for informed consent; 5、 Resume of principal investigator:<br>6、 List of researchers and division of responsibilities:		
voting results	Agree with 2 votes; Make necessary amendments ~~and agree~~ to make Tickets; disagree ______ Ticket: ~~ticket.~~ necessary amendments. Re-examination ticket: terminate or suspend the ______ ~~approved test.~~		
Review decision	Oral consent	Agree with the necessary amendments.	Re-trial after making necessary changes in the mouth.
	Mouth disagreement	To terminate or suspend the approved test.	

Review opinion

According to the National Health and Family Planning Commission's Measures for Ethical Review of Biomedical Research Involving People (2016) and SFDA's Clinical Medicine

Quality Management Standard for Bed Tests (2003), Quality Management Standard for Clinical Trials of Medical Devices (2016), WMA "H"

The ethical principles of Erxin Declaration and CIOMS International Ethical Guidelines for Human Health Research (2016) were reviewed by this Ethics Committee and agreed to carry out this research according to the approved clinical research plan, informed consent and recruitment materials.

Please follow the GCP principle and the scheme approved by the Ethics Committee to carry out clinical research and protect the health and rights of the subjects. In case of any of the following situations during the project, it is necessary to apply to/report to the Ethics Committee in writing in time:

1) Please submit an amendment review application in time for any modification of clinical research scheme, informed consent and recruitment materials.

Please;

2) To change the principal investigator, please submit the amendment review application in time;

3) In case of serious adverse events, please submit the serious adverse events report in time;

4) In case of any situation that may significantly affect the test or increase the risk of the subjects, please submit a written report in time:

5) In case of violation of the plan, please submit the report of violation of the plan in time;

6) To suspend or terminate the clinical research in advance, please submit the suspension/termination report in time.

<table>
<tr><td colspan="2">The Ethics committee wilt conduct a follow-up review of this project, and the applicant/sponsor is requested to submit a research progress report one month before the deadline according to the annual/regular follow-up review frequency specified by the Ethics Committee.

After the completion of this project, please submit a closing report to this ethics committee.

If the project fails to start clinical research within one year from the start date of the approval, the wooden approval is invalid and an application for ethical review needs to be re-submitted.</td></tr>
<tr><td>Annual/periodic follow-up review frequency</td><td>0 3 months 0 6 months 212 months Others. _ _ _ _
Please submit the research progress report before October 31, 2025.</td></tr>
<tr><td>term of validity</td><td>October 31st, 2024-0ctober 31st, 2025</td></tr>
<tr><td>Chairman/Deputy Chairman
(signature)</td><td></td></tr>
<tr><td colspan="2">邯郸市中心医院科研伦理委员会</td></tr>
<tr><td colspan="2">日期：2024年10月31 B</td></tr>
</table>

Note: Please use ¹"¹ ■" ; This document needs to be printed on both sides.'
